# Mediated, moderated and direct effects of country of residence, age, and gender on the cognitive and social determinants of adolescent smoking in Spain and the UK: a cross-sectional study

**DOI:** 10.1186/1471-2458-9-173

**Published:** 2009-06-04

**Authors:** Wolfgang A Markham, Maria Luisa Lopez, Paul Aveyard, Pablo Herrero, Christopher Bridle, Angel Comas, Anne Charlton, Hywel Thomas

**Affiliations:** 1 School of Health and Social Studies, University of Warwick, Coventry, CV4 7AL, UK; 2 Department of Preventive Medicine, Faculty of Medicine, University of Oviedo, 33006 Oviedo, Spain; 3 Department of Primary Care & General Practice, University of Birmingham, Birmingham B15 2TT, UK; 4 Health Sciences Research Institute, Warwick Medical School, University of Warwick, Coventry, CV4 7AL, UK; 5 Department of Epidemiology and Health Sciences, University of Manchester, Manchester, M13 9PT, UK; 6 School of Education, University of Birmingham, Birmingham B15 2TT, UK

## Abstract

**Background:**

European trans-national adolescent smoking prevention interventions based on social influences approaches have had limited success. The attitudes-social influences-efficacy (ASE) model is a social cognition model that states smoking behaviour is determined by smoking intention which, in turn, is predicted by seven ASE determinants; disadvantages, advantages, social acceptance, social norms, modelling, perceived pressure, self-efficacy. Distal factors such as country of residence, age and gender are external to the model. The ASE model is, thus, closely related to the Theory of Planned Behaviour. This study assessed the utility of the ASE model using cross-sectional data from Spanish and UK adolescents.

**Methods:**

In 1997, questionnaires were simultaneously administered to Spanish (n = 3716) and UK adolescents (n = 3715) who were considered at high risk of smoking. Participants' age, gender, smoking intentions and ASE determinant scores were identified and linear regression analysis was used to examine the mediated, moderated and direct effects of country of residence, age and gender on participants' smoking intentions.

**Results:**

All UK participants were aged 12 or 13 and most Spanish participants were aged between 12 and 14 (range 12–16 years). Amongst 12 and 13 year olds, regular smoking was more common in Spain. Almost half the participants were female (47.2% in Spain; 49.9% in the UK). Gender did not vary significantly according to age.

The distribution of ASE determinant scores varied by country and predicted intention. The influence of each ASE determinant on intention was moderated by country. Country had a large direct influence on intention (1.72 points on a 7 point scale) but the effects of age and gender were mediated by the ASE determinants.

The findings suggest resisting peer pressure interventions could potentially influence smoking amongst UK adolescents but not Spanish adolescents. Interventions that promote self-efficacy, on the other hand, would possibly have a greater influence on smoking amongst Spanish adolescents.

**Conclusion:**

The ASE model may not capture important cultural factors related to adolescent smoking and the relative contribution of particular ASE determinants to adolescent smoking intentions may differ between countries. Future European trans-national adolescent smoking prevention programmes may benefit from greater undestanding of country-level cultural norms.

## Background

Recent European Union policy statements advocate the development of trans-national adolescent smoking prevention interventions [1]. However, these initiatives are rare and the outcomes, to date, are variable [2,3]. The attitudes-social influences-efficacy or ASE model [4] is a social cognition model which could potentially underpin effective European trans-national teenage smoking prevention interventions. It explains adolescent smoking and adult smoking cessation in the Netherlands [4,5] and underpinned three successful Dutch adolescent smoking prevention trials [6-8]. However, there is little evidence regarding the utility of the ASE model and this study aims to contribute to the evidence. To our knowledge, no study, has examined the ability of the ASE model or the closely related Theory of Planned Behaviour (TPB) [9] to predict between-country smoking intentions of European adolescents in the way we outline. This study focuses on adolescents in Spain and the UK where adolescent smoking prevalence is relatively high compared with other European countries [10], but the findings have implications for theoretical development and future trans-national teenage smoking prevention interventions which are discussed.

The ASE model was developed from the Theory of Reasoned Action [11] and Bandura's Social Cognitive Theory [12], and purports to describe the predictors of volitional behaviour such as smoking [4,5,13]. According to the ASE model, future behaviour is determined by and closely related to intention. Intention in turn, is predicted by seven ASE determinants. The ASE determinants are, thus, posited to influence behaviour indirectly through intention. The ASE determinants are disadvantages, advantages, social acceptance, social norms, modelling, perceived pressure and self-efficacy. Disadvantages, advantages and social acceptance are described as attitudes. Disadvantages and advantages focus on the anticipated or actual experiences of the disadvantages and advantages of smoking. Social acceptance is a distinct sub-set of advantages and focuses on beliefs regarding the ability of smoking to facilitate social interactions. Social norms, modelling and perceived pressure are described as social influences. Social norms are respondents' beliefs regarding how influential people will feel about the respondent taking up smoking. Modelling refers to perceptions of the prevalence of smoking amongst individuals and groups who may potentially influence whether or not an adolescent smokes. Perceived pressure is experience of pressure, whether real or imagined, to smoke. Thus, social influences may be direct (social norms, perceived pressure) or indirect (modelling). Self-efficacy focuses on a person's beliefs regarding her/his ability to behave in the way that she/he wishes to behave with respect to smoking.

**Figure 1:**
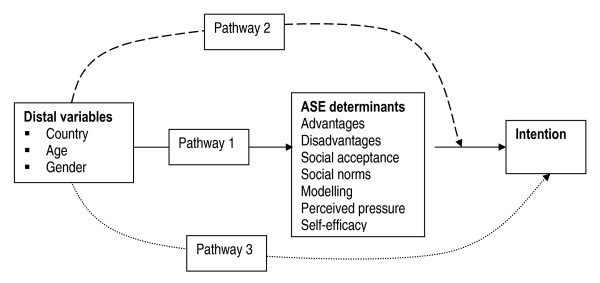
**The potential pathways through which country of residence, age and gender may influence intention**.

Distal factors, including country of residence, ethnicity and socio-demographic variables are external to the ASE model. De Vries et al. (1995) [4] posit that these distal influences are mediated by the ASE determinants (Figure 1, Pathway 1). Distal factors such as country of residence may also influence intention by moderating the influence of equivalent ASE determinant scores (Figure 1, Pathway 2). However, distal factors are postulated to have no direct influence on intention (Figure 1, Pathway 3).

The aim of this study was to investigate the influence of country of residence, age and gender on the adolescent smoking intentions. This investigation was driven by the ASE model and the assumptions underpinning the ASE model. We used Spanish and UK data collected in 1997 to examine the influence of country of residence, age and gender on adolescent smoking intentions via Pathway 1 (mediated effects), Pathway 2 (moderated effects) and Pathway 3 (direct effects). We have previously used the UK data to show that, as predicted by the ASE model, the distal influences of ethnicity (African-Caribbean, Indian, Pakistani and white), gender and socio-economic disadvantage on the smoking intentions of these UK teenagers were almost entirely mediated by the ASE determinants [14]. Adolescent smoking intentions are, according to the ASE model, closely related to smoking/non-smoking behaviour. Hence, for completeness we also identified actual smoking prevalence amongst the participants in both Spain and the UK.

## Methods

### Sampling

Our sampling and recruitment methods are described in detail elsewhere [14,15]. Briefly, the study focussed primarily on 12–14 year olds at high risk of smoking. In Spain, pupils (n = 3715) were recruited in the Asturias region and considered at high risk of smoking because they attended schools in towns of more than 50,000 residents, in which there was considerable tobacco advertising near schools [15]. In the UK, pupils were at high risk of smoking if they were socio-economically disadvantaged [16]. We recruited pupils from schools in three cites in the West Midlands where at least 30% of pupils' families received state benefits. These schools would be considered to serve disadvantaged communities. Of the 45 eligible mainstream secondary schools, 32 (71.1%) agreed to take part which resulted in pupils (n = 3716) participating in the project. For educational and logistical reasons, the Spanish and UK samples had slightly different mean ages and age ranges.

### Ethical approval

The data were collected in 1997 when it was not possible to gain ethical approval from any of the universities in Spain and the UK that employed the authors. The possibility of gaining ethical approval from UK universities is a relatively recent development. Most studies in the health field in the UK need National Health Service (NHS) ethics approval, but this was also not an option for us in 1997 because this study did not involve NHS staff or patients and the study was conducted in schools and, therefore, was not considered to be NHS territory. Hence, there was no body available to us that could consider any request for ethical approval. However, parents in Spain and the UK were asked in letters that also explained the aims of the study to contact the school if they wanted their children to opt out of the study. Pupils were also explicitly offered the chance to decline participation. Thus, we feel that how we collected the data was ethically appropriate and had we been able to seek ethical approval at the time of the study, it would have been granted.

### Questionnaire

Questionnaires were administered simultaneously in 1997 by researchers in Spain and by trained class teachers in the UK according to a protocol. In both Spain and the UK, no parent raised objections to the study and all pupils who were present on the days questionnaires were administered agreed to take part in the study. Students sealed their answered questionnaires in unmarked envelopes and were assured that their answers were confidential and that no one in the school would see their answers. The questionnaire was developed and validated in the Netherlands [4,8], and translated into English and Spanish and piloted [14,15]. The questions concerning behavioural intention and ASE determinants were of Likert scale type (Table 1). A mean score for each ASE determinant for each participant was obtained by adding the relevant individual question scores and dividing by the number of questions. Positive scores translate as promoting non-smoking. Questionnaire reliability was assessed in Spain, where a proportion (5%) of the sample answered the same questionnaire twice within a fifteen day interval. The κ for each question ranged from 0.74–1.00, indicating good/excellent test-retest reliability [17]. The Chronbach alphas for the attitudes and self-efficacy scales ranged from 0.63–0.92 indicating the constituent questions addressed the same construct. There is no reason to suppose the constituent questions of the social influences are related to each other because, for example, there is no reason to suppose that what a participant perceives her/his mother thinks will be related to what a participant perceives her/his best friend thinks.

**Table 1:** Summary of the questions pertaining to the ASE determinants and intention

ASE determinant	Question topics	Range and meaning	Cronbach's alpha
**Intention**	Do you want to be a smoker in the future	-3 definitely 0 don't know +3 definitely not	N/A

**Attitudes**			

Advantages	Smoking positively affects Taste When bored, When nervous	-3 strongly agree 0 uncertain +1 disagree	0.67(Spain) 0.85(UK)

Disadvantages	Smoking negatively affects ... General health Passive smoking Coughing Fitness Breathing Smoking being a silly thing to do	-1 disagree 0 uncertain +3 strongly agree	0.70(Spain) 0.82(UK)

Social acceptance	Smoking positively affects ... Getting on with friends, Joining in with other people, Meeting new people, Being teased	-3 strongly agree 0 uncertain +3 strongly disagree	0.81(Spain) 0.63(UK)

**Social influences**			

Social norms	What do the following people think about you smoking? Most people who are important to me Mother, Father, Brother (s), Sister(s), Friends Best friend	-3 definitely think I should smoke 0 uncertain +3 definitely think I should not smoke	N/A

Modeling 1	Do the following people smoke Mother Father Siblings, Best friend	-4 do smoke- 0 do not smoke	N/A

Modeling 2	How many of the following people smoke Friends, Classmates, Teachers	-4 most smoke -3 a lot smoke -2 half smoke -1 few smoke 0 none smoke	N/A

Perceived pressure	How often have you felt pressure to smoke from Mother, Father, Brother(s), Sister (s), Friends, Best friend, Class mates, Teachers, Relatives,	-4 very often 0 never	N/A

**Self-efficacy**	How easy is it not to smoke if you don't want to When with others who are smoking, When with friends who are smoking, When offered a cigarette, When parent offers a cigarette, When teased because you don't want a cigarette	-3 it would be very difficult to resist 0 uncertain +3 it would be very easy to resist	0.88(Spain) 0.92(UK)

### Assessment of smoking

Adolescents who regularly smoked at least one cigarette a week were considered regular smokers in this study [18]. Regular smoking was assessed through the standard question [18,19] that asks which of six descriptions best describes participants' smoking behaviour. These descriptions were 'I have never smoked', 'I have only ever tried smoking once', 'I used to smoke sometimes, but I never smoke a cigarette now', 'I sometimes smoke cigarettes now but I don't smoke as many as one a week', 'I usually smoke between one and six cigarettes a week' and 'I usually smoke more than six cigarettes a week'. Standard question responses were checked against a dichotomous recent smoking behaviour question. Recent smoking history was used to reclassify ex-smokers and occasional smokers from the standard question as regular smokers or non-smokers. Participants were omitted from the analysis if they gave inconsistent smoking behaviour answers (Spain 51 (1.5%); UK 137 (3.7%)).

### Data Analysis

We used linear regression analysis in SPSS 10.00 for Windows. All models were checked and showed no evidence of heteroscedasticity and evidence of multivariable normality. Missing single variables were included as dummy terms. Thus, for example, if age was missing then the model included a term which was age missing yes and the reference group was age not missing. We reran the analyses conducted below, excluding any participants who were not 12–14 years old. The results were similar, so only the analyses reported below are presented.

### The ability of the ASE model to predict smoking intentions, a within country analysis

We separated the data by country and linearly regressed the intention score on all ASE determinants, and then controlled for age and gender using dummy terms. We calculated the adjusted r^2 ^and the F test for the explanatory effect of ASE determinants alone, and the addition of age and gender.

### Are the influences of country of residence, age and gender on smoking intentions, mediated by the ASE determinants? A between country analysis

To examine whether the influences of country of residence, age and gender are mediated through the ASE determinants we pooled the Spanish and UK data and followed the method of Baron and Kenny [20] which involves four steps. Each of the following criteria should be satisfied in order to demonstrate mediation.

Step I Country of residence, age and gender (the independent variables) are correlated with smoking intention (the dependent variable).

Step II Country of residence, age and gender are associated with each ASE determinant.

Step III Each ASE determinant is related to smoking intention when country of residence, age and gender are controlled for.

Step IV Complete mediation is shown if, in Step III, controlling for the ASE determinants abolishes any residual influence of age, gender, and country of residence on intention.

According to Kenny et al. [21], Step I is not required if there is no theoretical justification for assuming a possible reverse causation. We believe there is no theoretical justification for assuming smoking intention could possibly influence country of residence or age or gender. Hence, we did not investigate reverse causation by examining the correlation between country of residence, age and gender and smoking intention.

#### Step II: Are country of residence, age and gender correlated with each ASE determinant?

For Step II we used linear regression to examine whether country, age and gender were associated with each individual ASE determinant. We examined whether the influence of country varied by age or gender using multiplicative interaction terms (country × age and country × gender) in each ASE determinant equation.

#### Steps III and IV: Is each ASE determinant related to intention and does this abolish the association of age, gender, and country of residence on intention?

The effects in Step III (Pathway 1) and Step IV (Pathway 3) were estimated using linear regression predicting intention with all the ASE determinants, country, age and gender entered. We added country × age and country × gender interaction terms to examine whether the direct effects of country were only apparent amongst one gender or age group. If Pathway 1 was important, each ASE determinant would predict intention. If Pathway 3 was important, age, gender, and country would have direct unmediated effects on smoking intention even though the model included all the ASE determinants.

### Does country of residence modify the influence of equivalent ASE determinant scores on smoking intention? A between country analysis

To determine whether country of residence modifies the influence of equivalent ASE determinant scores on smoking intention, in other words to determine whether Pathway 2 was important, we repeated Steps III and IV above and included multiplicative ASE determinant score × country interaction terms. Significant interaction effects imply the influence of equivalent ASE determinant scores on intention varied between countries. For ease of interpretation, we graphed the predicted effects of each ASE determinant on intention by country. The scores for all other ASE determinants bar the one being graphed were set at zero.

## Results

### Characteristics of the sample

Smoking status and demographic characteristics were tabulated by country (Table 2). Amongst 12 and 13 year olds, regular smoking was more common in Spain than the UK (Table 2).

**Table 2:** Demographic characteristics of the population in each country

Age		Spain	UK
Under 12			
12–12.99			
13–13.99		241 (6.5)	1243 (33.5)
14–14.99		982 (26.4)	2459 (66.2)
15–15.99		1303 (35.1)	
16–oldest		855 (23)	
Missing		331 (8.9)	
Gender		3 (0.1)	14 (0.4)
Females			
Males		1755 (47.2)	1839 (49.9)
Missing		1957 (52.7)	1846 (50.1)
		3 (0.1)	31 (0.8)
Smoking status			
<12	Males	0 (0.0%)	
	Females	1 (10.0%)	
12–12.99	Males	60 (12.0%)	40 (6.7%)
	Females	52 (9.7%)	41 (6.9%)
13–13.99	Males	84 (15.2%)	91 (7.8%)
	Females	69 (15.7%)	86 (7.3%)
14–14.99	Males	22 (19.0%)	
	Females	22 (23.9%)	
15–15.99	Males	7 (43.8%)	
	Females	0 (0.0%)	
>16	Males	9 (23.1%)	
	Females	4 (16.0%)	
All age groups	Males	182 (14.7%)	131 (7.4%)
	Females	148 (13.4%)	127 (7.1%)
Total		3715	3716

### The ability of the ASE model to predict smoking intentions, a within country analysis

In Spain, the adjusted r^2 ^was 0.36 when the ASE determinants were entered alone (F = 298.1, df = 7, 3702, p < 0.001). This increased trivially to 0.37 on the addition of age and gender (F = 8.6, df = 5, 3697, p =< 0.001). In the UK, the adjusted r^2 ^was 0.29 when the ASE determinants were entered alone (F = 206.6, df = 7, 3520, p < 0.001). Age and gender did not add significantly to the explanatory power of the UK model. (F = 2.8, df = 2, 3518, p = 0.064).

### Are the influences of country of residence, age and gender on smoking intentions, mediated by the ASE determinants? A between country analysis

#### Step II: Are country of residence, age and gender correlated with each ASE determinant?

Country had a statistically significant and often marked influence on each ASE determinant (Table 3). Excepting modelling, all the ASE determinants were more anti-smoking for Spanish adolescents than for English adolescents. There were also significant interactions with gender in Spain for all but one ASE determinant (self-efficacy). Although not shown, for every ASE determinant, age was predictably negatively associated with more anti-smoking effects.

**Table 3:** Regression equation for the influence of age, gender, and country on each ASE determinant

	Advantages	Disadvantages	Social acceptance	Social norms	Modelling	Perceived pressure	Self-efficacy
	B (95%CI)	B (95%CI)	B (95%CI)	B (95%CI)	B (95%CI)	B (95%CI)	B (95%CI)
**Country, age** **and gender**†							
UK females	0.00	0.00	0.00	0.00	0.00	0.00	0.00
UK males	0.00	-0.06	0.06	-0.14	-0.02	-0.13	0.07
Spanish females	0.19	0.08	1.64	0.09	-0.16	0.17	1.83
Spanish males	0.27	0.28	1.34	0.24	-0.01	0.29	1.92
**Main effects** **and interaction** **terms**							
Gender							
Male	0.00 (-0.05–0.06)	-0.06 (-0.12–-0.01)*	0.06 (-0.01–0.12)	-0.14 (-0.20–-0.08)***	-0.02 (-0.07–0.04)	-0.13 (-0.16–-0.10)***	0.07 (-0.03–0.17)
Country							
Spain	0.19 (0.12–0.26)***	0.08 (0.01–0.16)*	1.64 (1.55–1.74)***	0.09 (0.01–0.18)*	-0.16 (-0.23–-0.09)***	0.17 (0.12–0.21)***	1.83 (1.70–1.96)***
Gender * country							
Males * Spain	0.08 (0.00–0.15)*	0.20 (0.12–0.28)***	-0.30 (-0.40–-0.20)***	0.15 (0.06–0.23)**	0.15 (0.07–0.22)***	0.12 (0.07–0.16)***	0.09 (-0.04–0.23)

* < 0.05

** < 0.01

*** < 0.001

† Combines country main effects, gender main effects, and country × gender interactions

#### Steps III and IV: Is each ASE determinant associated with intention and does this abolish the association of age, gender, and country of residence on intention?

Each ASE determinant, except social acceptance, had significant main effects on intention (Table 4) supporting Pathway 1 (Figure 1). This indicated that differences between countries in smoking intentions are at least partially mediated by differences in the distributions of the ASE determinant scores.

**Table 4:** ASE determinants as predictors of intention

ASE determinant†	B (95%CI)
Advantages	0.34 (0.29–0.40)***
Advantages Spain	-0.15 (-0.23–-0.07)***
Disadvantages	0.31 (0.27–0.36)***
Disadvantages Spain	0.13 (0.06–0.21)**
Social acceptance	0.01 (-0.03–0.06)
Social acceptance Spain	0.01 (-0.07–0.05)
Social norms	0.14 (0.10–0.18)***
Social norms Spain	0.11 (0.03–0.18)**
Modelling	0.33 (0.27–0.39)***
Modelling Spain	-0.14 (-0.23–-0.06)**
Perceived pressure	0.42 (0.34–0.50)***
Perceived pressure Spain	-0.46 (-0.65–-0.27)***
Self efficacy	0.13 (0.10–0.15)***
Self efficacy Spain	0.15 (0.11–0.20)***

* < 0.05

** < 0.01

*** < 0.001

† Reference group

There was very strong evidence for Pathway 3 (Figure 1) arising from country, but not age or gender. When all ASE scores were controlled simultaneously, there was a large difference (1.72 points on a 7 point scale) in intention to smoke in Spain compared to the UK. However, the effects were slight for age (0.07 points on a 7 point scale for 12 and 14 year olds) and gender (0.12 points on a 7 point scale). Thus, the influences of age and gender on smoking intentions amongst the Spanish and UK adolescents were almost entirely mediated by the ASE determinants. However, the effects of country on smoking intentions were only partially mediated by the ASE determinants.

### Does country of residence modify the influence of equivalent ASE determinant scores on smoking intention? A between country analysis

**Figure 2:**
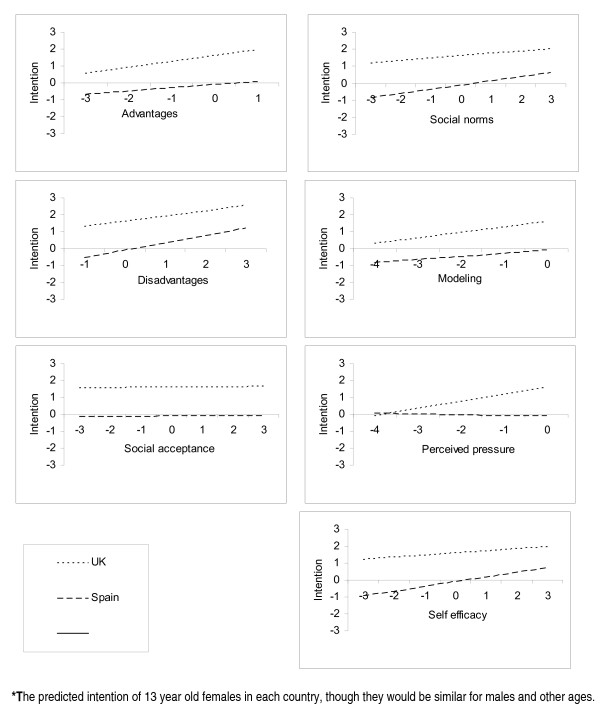
Graphs showing influence of ASE determinants on intention to smoke adjusted for sociodemographic factors*.

The influence of each ASE determinant on intention varied considerably between countries (Table 4; Figure 2) supporting Pathway 2 (Figure 1). Interactions were the norm, not the exception (Table 4). There are five salient points contained within Table 4 and Figure 2. First, for nearly all possible ASE scores, English adolescents had more anti-smoking intentions than Spanish adolescents. Second, perceived pressure had no influence on Spanish adolescents. Third, self-efficacy had relatively little influence on intention in the UK, compared to Spain. Fourth, the effects on intention of differences in the attitudes scores (advantages, disadvantages and social acceptance) were broadly similar in both countries as judged by the gradients of the lines in Figure 2. However, the predictive effects of equivalent social influences (social norms, modelling and perceived pressure) and self-efficacy scores on intention varied importantly between countries. Fifth, social acceptance had no significant country interactions.

## Discussion

This study indicates the influence of country of residence on adolescent smoking intentions was only partially mediated through the ASE determinants. Country also modified the influences of similar adolescent cognitions on smoking intentions. Contrary to the predictions of the ASE model, country appeared to have a large direct influence on adolescent smoking intentions. In our within-country investigation of the same UK participants, we found the external influences of ethnicity (African-Caribbean, Indian, Pakistani and white) gender and socio-economic disadvantage on adolescent smoking intentions were almost entirely mediated by the ASE determinants [14]. Being a white boy had a small direct influence on intention. Otherwise ethnicity, gender and socio-economic disadvantage had no direct effects on smoking intentions. Additionally, the external influences of ethnicity, gender and socio-economic disadvantage did not modify the predictive effects of equivalent ASE determinant scores on intention. Thus, the findings of the between-country study reported here are very different to the findings of our within-country study [14]. The findings of the between-country study reported here may have implications for theoretical development and the future development of pan-European adolescent smoking prevention interventions.

### Implications of the findings for theoretical development

Why might the effects of equivalent social influences (social norms, perceived pressure and modelling) scores on adolescent smoking intentions have varied by country? First, it is conceivable that some role models such as pop singers were excluded from the salient role set used to assess modelling and this would affect the relative influence of modelling on smoking intentions in each country. However, it seems unlikely that we omitted salient actors from the role sets used to assess social norms and perceived pressure. Second, if equal weighting of all sources of social influence was broadly appropriate in one country, but not in the other, this would create the apparent differences between countries regarding the influence of social norms, modelling and perceived pressure on intention to smoke. Third, one influence, for example, mothers, may have the greatest influence on adolescents in both countries. However, equal weighting of all the sources of the social influence indices may mask the influence of mothers. Thus, both countries may have a similar modelling index but one has a high prevalence of maternal smoking, the social influence with the greatest influence on adolescent smoking intentions, while the other has a low prevalence of maternal smoking.

One of the most striking findings was the large difference between countries in self-efficacy. A typical Spanish adolescent not only has greater self-efficacy than a typical English adolescent but self-efficacy also has a greater influence on Spanish adolescents' smoking intentions. This could have arisen if the meanings of the social situations used to measure self-efficacy in this study vary between countries which may influence beliefs about how much individual control may be exerted by respondents [12].

Differential weighting of the ASE determinants does not threaten the validity of the ASE model. However, if the ASE model allows that the relative importance of the ASE determinants varies with respect to adolescent smoking intentions, then this implies the relative importance is determined by some organizing construct specific to the overarching culture of individual countries which is currently missing from the model. We included country × age and country × gender interactions, but these were not significant. Additionally, previous analysis of the UK data indicated that participants' ethnicity, age socio-economic status and gender did not moderate the influence of the predictive effects of ASE determinants on smoking intentions [14]. These findings suggest that the organizing construct is specific to individual countries and understood similarly by all adolescent sub-groups within countries based on ethnicity, age, socio-economic status, and gender.

Country of residence had a large direct influence on intention (1.72 points on a 7 point scale). Previous analysis of the UK data indicted that participants' socio-economic status and ethnicity did not independently directly influence smoking intentions [14]. This suggests that between-country variation in both the socio-economic status and ethnicity of the participants does not underpin the additional explained variance (independent effect) of country on adolescent smoking intentions. Two possible explanations for the direct influence of country on adolescent smoking intentions are related to affective and moral beliefs which are not included in the model. First, Connor and Armitage (1998) [22] distinguish between the influences of instrumental, affective and moral beliefs on attitudes. Our study only measured attitudes that focus on instrumental beliefs. Second, Ajzen proposed that moral norms could operate alongside other determinants of intention and directly influence intention when decisions have ethical or moral dimensions [9]. The concept of moral norms has been extended to cover personal norms, where the use of a moral framework is problematic though not entirely redundant, such as with adolescent smoking [23-25]. That is, individuals have an inter-related set of values, closely allied to self-identity. Not only are some value systems/self-identities more compatible with smoking than others, but these value systems/identities and their associations with smoking or non-smoking may vary by country. If this is the case, whatever the nature of these value systems/identities, they appear to be shared by most English adolescents in our sample regardless of ethnicity but are distinct from those shared by Spanish adolescents in our sample. Understanding of the relationships between value systems/identities and adolescents' smoking intentions is relatively underdeveloped. However, moral beliefs have been shown to be a major cause of differences in smoking prevalence amongst UK Bangladeshi female and male adolescents [24].

Viewing cigarettes as a social handicap or social facilitator did not predict intention in either country. If confirmed, social acceptance could be omitted from the model.

### Implications of the findings for European trans-national adolescent smoking prevention interventions

Adolescent smoking prevention interventions that are driven by the ASE model aim to change the cognitions underpinning the ASE determinants in order to alter behavioural intention and, thus, future smoking. Differential weighting of ASE determinants between countries has two implications for the development of effective trans-national adolescent smoking prevention programmes. First, the usefulness of each ASE determinant as a predictor may vary according to country. A pan-European intervention to resist peer pressure, for example, would be predicted *a priori *to be effective. However, our results suggest that although this type of intervention could potentially influence UK adolescent smoking intentions, the same type of intervention would have little effect on Spanish adolescent smoking intentions. Interventions that aimed to promote self-efficacy, on the other hand, would be more likely to have a greater influence on Spanish adolescent smoking intentions than on UK adolescent smoking intentions. Second, given the large variations between countries in the predictive effects of particular ASE determinants, understanding the possible country specific organizing constructs highlighted above may be important for the effective implementation of trans-national adolescent smoking prevention programmes. Without this additional understanding, trans-national adolescent smoking prevention initiatives may fail, but the overall results may hide important successes within some countries. Additionally, we have argued that value systems/identities and some attitudes that are underpinned by salient affective and moral beliefs may be important influences on adolescents' smoking intentions, but understanding of these is poorly developed. Given the large influence these variables may potentially have, we suggest their examination could be an important component of future trans-national teenage smoking prevention programmes.

European trans-national adolescent smoking prevention initiatives are rare and have had variable results [2,3]. A life-skills smoking intervention for German speaking pupils in Austria, Denmark, Germany and Luxembourg had little effect on current smoking [3]. The ESFA adolescent smoking prevention intervention study was conducted in six countries (Denmark, Finland, Portugal, Spain, the Netherlands and the UK) and was underpinned by the ASE model [2]. The interventions had an apparent moderate effect on regular smoking in some countries after twenty four and thirty months. However, this study had methodological problems including the non-randomized nature of some data, potential biases in data collection and analysis, very high attrition rates, relatively high exclusion rates and adjustment for covariates in the analyses in order to increase power. The interventions used in the ESFA project were tailored to the cultural circumstances of each participating European country. However, this tailoring was based on practical and logistical concerns rather than theoretical considerations and appropriateness. Spain and the UK participated in the ESFA project but it is unclear how the ASE model informed the development of the interventions used in these countries. The ESFA project results may have arisen because cultural factors were not adequately accounted for [26]. Given our results, it is perhaps unsurprising the ESFA project found the English and Spanish interventions had very different effects on adolescent smoking outcomes.

### Study limitations

In this study, the ASE model explained 37% of the variance of intention in Spain and 29% in the UK, which Sutton describes as explaining a medium-high percentage of the variance [27]. However, our study was cross-sectional so we can only say that the ASE determinants are associated with adolescent smoking intentions. Prospective studies have however, confirmed the predictive ability of the ASE model [6]. Nonetheless, any conclusions concerning the ability of the ASE model to predict adolescent smoking intentions based on our findings should be regarded as tentative.

There are two possible biases that could have caused spurious differences in smoking intentions between countries and thus, the large unmediated effect of country. The first bias relates to the translation of the questionnaire from the original Dutch questionnaire. The intended meaning of the questions may not have been adequately reflected in the British and Spanish translated versions. This bias is an unlikely explanation for the differences in intention between countries, however, because the questionnaires showed good reliability in each country. The second bias could arise if participants' interpretations of the questions vary between individuals or between groups based on age, gender, or country. Thus, even if the translations were completely accurate, participants' interpretations could depend on the social context of their lives. However, bias is an unlikely cause of the direct unmediated effect of country of residence on smoking intentions because the differences between the predicted maximum and minimum intention scores arising from each ASE determinant (Figure 2) were less than the unexplained difference in intention between countries.

Behavioural intention was assessed using one question, which is commonly the case [27]. However, two meta-analyses concluded that behavioural intention is a fairly robust construct and the type of measure of behavioural intention does not greatly influence the predictive ability of behavioural intention [28,29].

The ASE model and the closely related TPB are currently still being used by researchers and health promoters as a basis for the development of adolescent smoking prevention interventions in many countries. Both the ASE model and the TPB have similar underpinning assumptions and these assumptions are currently commonly considered to be valid. The aim of the investigation was to test the validity of the underpinning assumptions of the ASE model. We used data that were collected in 1997 and are thus, relatively old. Nevertheless the ASE model should not, in theory, be dependent on the age of the data. In other words there is no reason to assume that a theoretical model such as the ASE model should lose its applicability over a ten year period. The ASE model and its underpinning assumptions are valid for each country [14]. However, this investigation indicates that the importance of the ASE determinants varies according to country and the ASE model may fail to capture important cultural factors.

## Conclusion

This study indicates that social cognition models such as the ASE model may predict adolescent smoking within countries but the relative contribution of particular antecedents may differ between countries. Thus, pan-European adolescent smoking prevention interventions may benefit from the identification of the psychosocial determinants that best predict smoking intention in each country in order to tailor smoking prevention interventions to the needs of adolescents in the different countries. Additionally, social cognition models such as the ASE model may not currently capture important cultural factors that influence adolescent smoking intentions. Further cross-cultural investigations of the long-term relevance of cultural norms for adolescent smoking may also reveal important information for future adolescent smoking prevention programmes.

## Competing interests

The authors declare that they have no competing interests.

## Authors' contributions

PA and WM conceived of the investigation, devised the analysis and wrote the first drafts of the paper. WM, ML, PH, AC, AC and HT devised the original study, collected the data and worked on drafts of the manuscript. CB helped with the analysis and worked on drafts of the manuscript. All authors read and approved the final manuscript.

## Pre-publication history

The pre-publication history for this paper can be accessed here:


